# Supplementary material for: Large Data Set Analysis Reveals Structural Origin of Peptide Collisional Cross Section Bimodal Behavior
Source: J Am Soc Mass Spectrom. 2025 Dec 21;37(1):279–88. doi: 10.1021/jasms.5c00325 (PMC12784393; doi:10.1021/jasms.5c00325)
Supplement: Supplementary file 1 [file js5c00325_si_001.pdf]

Supporting Information: Large Dataset Analysis Reveals Structural Origin of Peptide Collisional Cross Section Bimodal Behavior

Allyn M. Xu,[†] Dániel Szöllősi,[‡,¶] Helmut Grubmüller,[‡] and Oded Regev[*,†]

†*Computer Science Department, Courant Institute of Mathematical Sciences, New York University, New York, NY 10012, USA*

‡*Department of Theoretical and Computational Biophysics, Max Planck Institute for Multidisciplinary Sciences, Göttingen D-37077, Germany*

¶*Current address: Research Service Center, Vienna University of Economics and Business, Vienna 1020, Austria*

E-mail: regev@cims.nyu.edu

Supplementary Figures

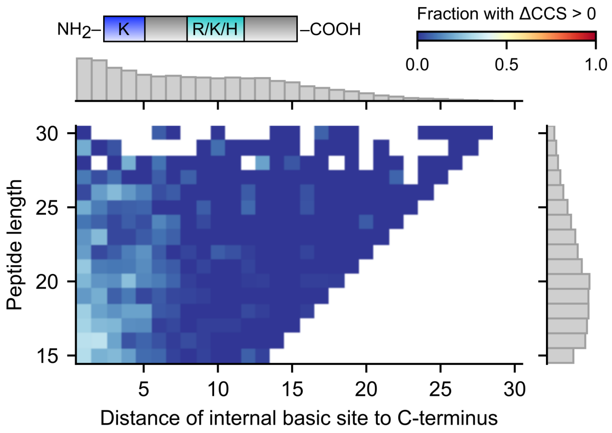

Figure S1: **CCS bimodality for LysN peptides (related to Fig. 1C).** 2D histogram of peptide length versus distance (in amino acids) of internal basic site to C-terminus for charge 3^+ non-Nt-acetylated LysN peptides with three basic sites, colored by fraction of peptides in bin with ΔCCS > 0. Bins with less than 8 peptides were excluded. Marginal density histograms on right and top axes, respectively (grey boxes). Cartoon of peptide shown at the top, with internal basic site colored in teal, and N-terminal lysine colored in blue.

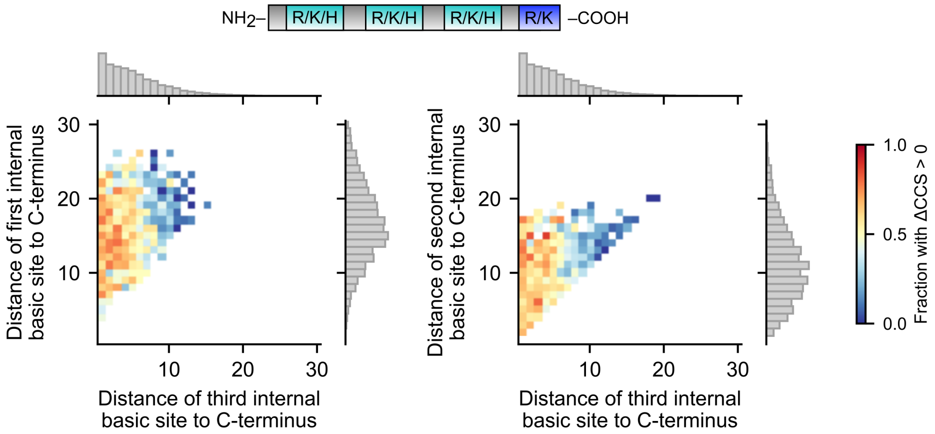

Figure S2: **CCS bimodality in peptides with five basic sites (related to Fig. 2).** 2D histogram of the distance of the first versus third internal basic site (left) and of the second versus third internal basic site (right) from the C-terminus for charge 3^+ non-Nt-acetylated tryptic peptides with five basic sites, colored by fraction of peptides in bin with ΔCCS > 0. Bins with less than 8 peptides were excluded. Marginal density histograms on right and top axes, respectively (grey boxes). Cartoon of peptide shown at the top, with internal basic site colored in teal, and C-terminal arginine or lysine colored in blue.

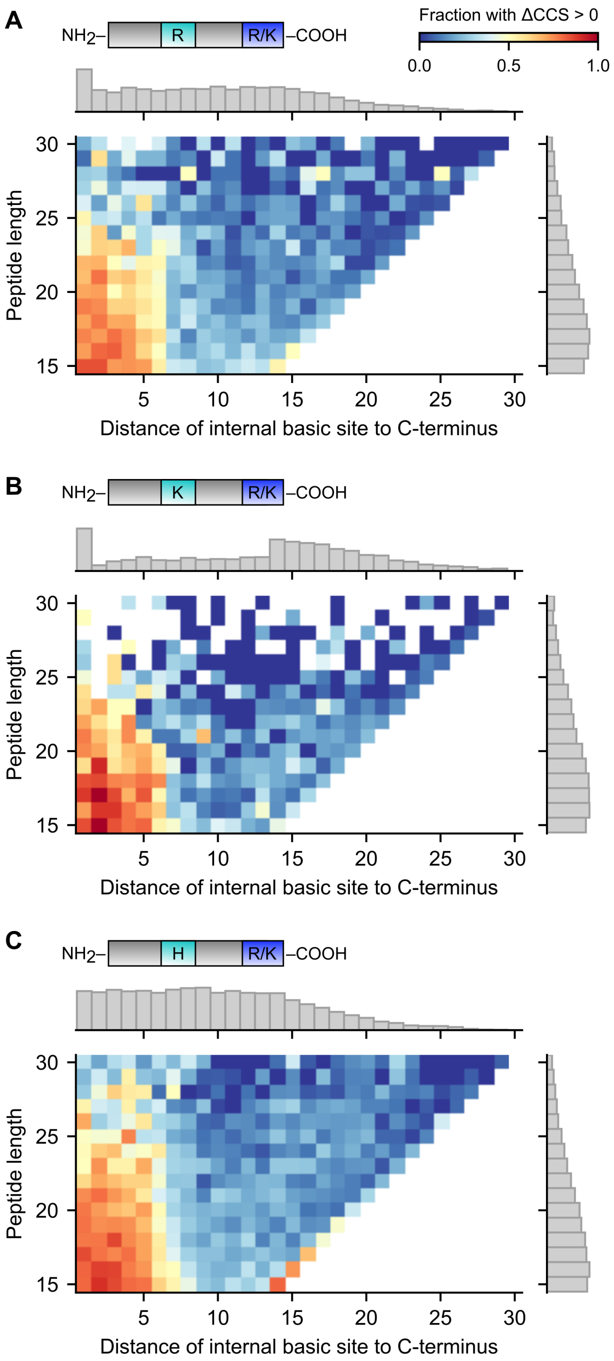

Figure S3: **CCS bimodality with a specific internal basic site (related to Fig. 1C).** 2D histogram of peptide length versus distance (in amino acids) of internal basic site to C-terminus for charge 3^+ non-Nt-acetylated tryptic peptides with three basic sites whose internal basic site is a arginine **(A)**, lysine **(B)**, and histidine **(C)**, colored by fraction of peptides in bin with ΔCCS > 0. Right-most diagonal corresponds to the internal basic site being at the N-terminus. Bins with less than 3 peptides were excluded. Marginal density histograms on right and top axes, respectively (grey boxes). Cartoon of peptide shown at the top, with internal basic site colored in teal, and C-terminal arginine or lysine colored in blue.

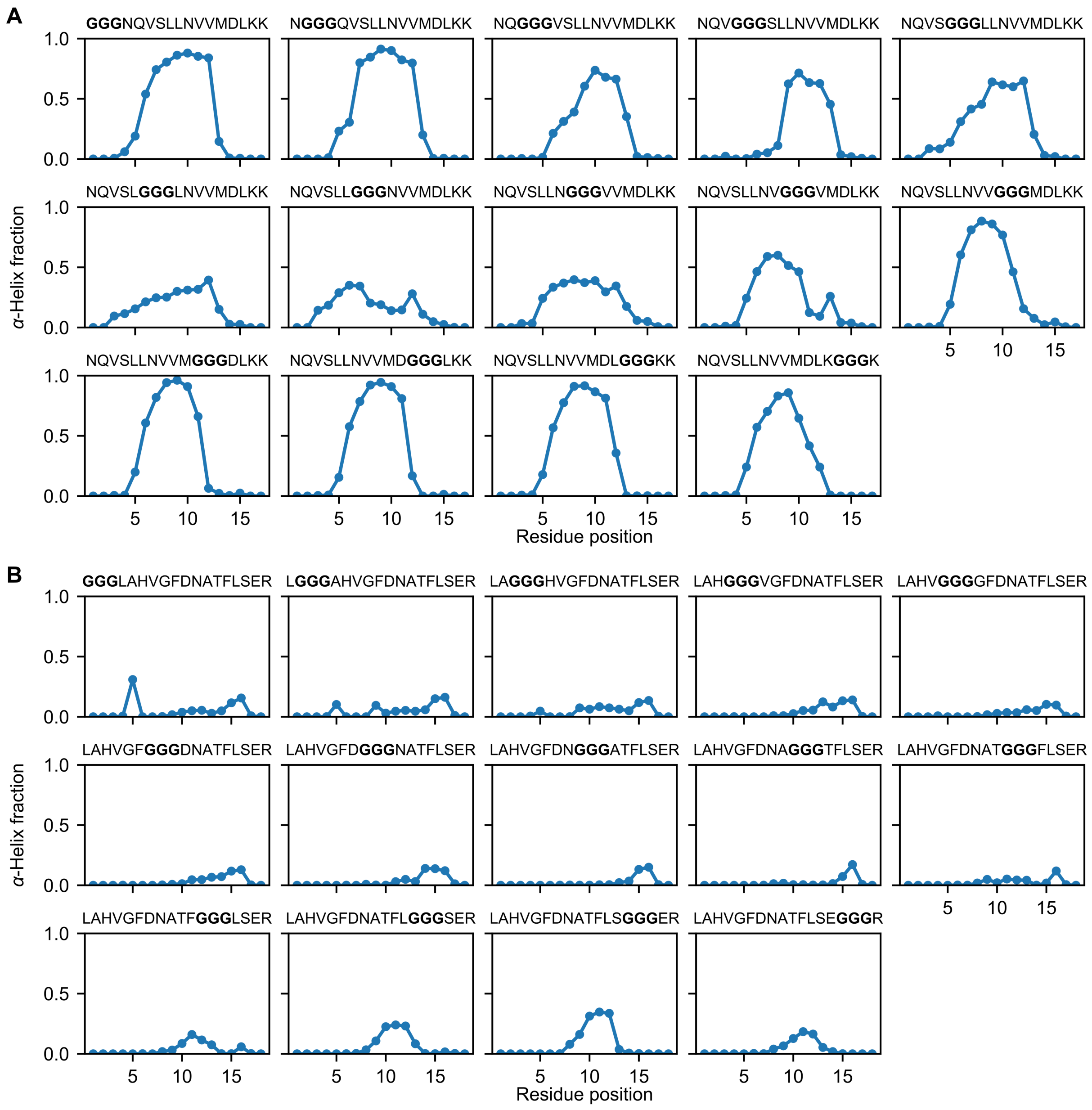

Figure S4: α-**helix fraction by residue.** Plot showing the fraction of MD simulation runs where a given residue was involved in an α-helix, for each simulated peptide. Plots corresponding to peptides in the high mode series grouped in (A) and the low mode in (B). Glycine triplet is shown in bold.

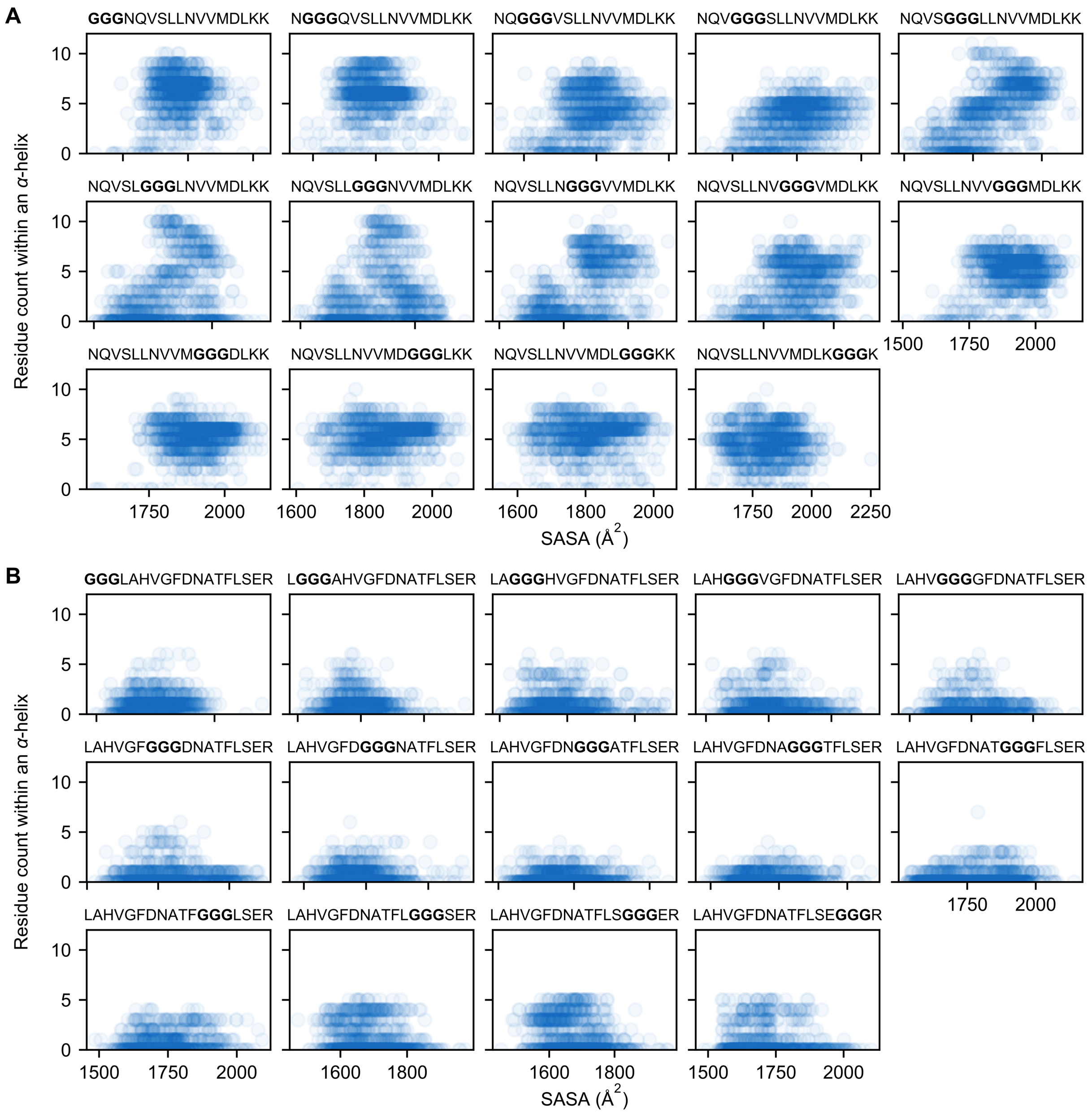

Figure S5: **Residue count within an α-helix vs SASA.** Scatter plot of the number of residues within an α-helix versus SASA across MD simulation runs, for each simulated peptide. Each plot contains 1000 points, one for each MD simulation run ($\alpha = 0.05$). Plots corresponding to peptides in the high mode series grouped in (A), and those in the low mode series in (B). Glycine triplet is shown in bold.
